# Two-Photon Polymerisation 3D Printing of Microneedle Array Templates with Versatile Designs: Application in the Development of Polymeric Drug Delivery Systems

**DOI:** 10.1007/s11095-020-02887-9

**Published:** 2020-08-27

**Authors:** Ana Sara Cordeiro, Ismaiel A. Tekko, Mohamed H. Jomaa, Lalitkumar Vora, Emma McAlister, Fabiana Volpe-Zanutto, Matthew Nethery, Paul T. Baine, Neil Mitchell, David W. McNeill, Ryan F. Donnelly

**Affiliations:** 1 School of Pharmacy, Queen’s University Belfast, Belfast, UK; 2 Department of Pharmaceutics and Pharmaceutical Technology, Faculty of Pharmacy, Aleppo University, Aleppo, Syria; 3 School of Electronics, Electrical Engineering and Computer Science, Queen’s University Belfast, Belfast, UK

**Keywords:** 3D printing, dissolving, hydrogel-forming, microneedle array, two-photon polymerisation

## Abstract

**Purpose:**

To apply a simple and flexible manufacturing technique, two-photon polymerisation (2PP), to the fabrication of microneedle (MN) array templates with high precision and low cost in a short time.

**Methods:**

Seven different MN array templates were produced by 2PP 3D printing, varying needle height (900–1300 μm), shape (conical, pyramidal, cross-shaped and with pedestal), base width (300–500 μm) and interspacing (100–500 μm). Silicone MN array moulds were fabricated from these templates and used to produce dissolving and hydrogel-forming MN arrays. These polymeric MN arrays were evaluated for their insertion in skin models and their ability to deliver model drugs (cabotegravir sodium and ibuprofen sodium) to viable layers of the skin (ex vivo and in vitro) for subsequent controlled release and/or absorption.

**Results:**

The various templates obtained with 2PP 3D printing allowed the reproducible fabrication of multiple MN array moulds. The polymeric MN arrays produced were efficiently inserted into two different skin models, with sharp conical and pyramidal needles showing the highest insertion depth values (64–90% of needle height). These results correlated generally with ex vivo and in vitro drug delivery results, where the same designs showed higher drug delivery rates after 24 h of application.

**Conclusion:**

This work highlights the benefits of using 2PP 3D printing to prototype variable MN array designs in a simple and reproducible manner, for their application in drug delivery.

**Electronic supplementary material:**

The online version of this article (10.1007/s11095-020-02887-9) contains supplementary material, which is available to authorized users.

## Introduction

The use of microneedle (MN) arrays to overcome the limitations of conventional drug delivery has become a growing research field in the past decades. These minimally invasive structures, usually comprising multiple micron-sized needles, have shown abilities to deliver a variety of molecules and nanoparticles, for therapeutic and vaccination purposes (1,2). Furthermore, these innovative devices can also be used to collect skin interstitial fluid, expanding their potential applications to other fields such as diagnostics or drug monitoring (3–5). Throughout the years, MN arrays have been fabricated using a variety of materials, including silicon, metal, ceramics, glass and, more recently, multiple natural and synthetic polymers. For this purpose, researchers have used manufacture techniques mainly emerging from the microelectronics industry, such as reactive ion etching, lithography, electroplating, laser cutting, injection moulding and micromoulding (2).

Polymeric microneedle arrays have particular interest for the pharmaceutical industry, since they generally present good biocompatibility, degradability and mechanical properties at a reduced cost. Additionally, the disposal of these arrays does not generate any sharp waste, as they can be mechanically or chemically destroyed, or even dissolved by the interstitial fluid in the skin in the case of water-soluble polymers (6–8). The majority of polymeric MN arrays are manufactured through mould-based techniques, such as casting, hot embossing, injection moulding, drawing lithography or laser micromachining, among others (2). Micromoulding-based manufacture of MN arrays implies the use of a mould, commonly made from poly (dimethylsiloxane) (PDMS), to replicate the MN array structure. These moulds are usually obtained from silicon or metal master templates, which in turn are fabricated by lithography, etching or laser-based techniques (2,9). However, these may prove restrictive in the fabrication of complex MN array designs and geometries.

More recently, the wide range of additive manufacturing (or 3D printing) technologies available in academia and industry have opened an interesting new field of research for the manufacturing of MN arrays. Three-dimensional (3D) printing is a pioneering technology introduced in the 1980s which uses a model created by Computer-Aided Design (CAD) to manufacture a physical object through consecutively creating and adding layers (10). As it minimises manufacturing time and enables the reproducible production of highly complex objects, 3D printing has naturally attracted the attention of pharmaceutical, biomedical and material scientists (10–12). The different technologies available within the 3D printing scope include inkjet printing, fused deposition modelling and photopolymerisation, with the latter being the most common choice for MN array fabrication. This technique is based on the ability of a light source (commonly a laser beam) to selectively polymerise photo-sensitive materials, curing each layer as the light is emitted. In general, a digitally controlled printing head emits light to selective points of a building platform immersed in uncured resin, forming consecutive layers of newly cured solid polymer (10). The two most common forms of photopolymerisation are stereolithography (SLA) and digital light processing (DLP), with various examples of their applications available in the literature (13–18).

Two-photon polymerisation (2PP) has been developed in recent years to enable the manufacturing of elaborate structures in the micro and nanoscale (19). In this specific technology, ultrashort laser pulses from a near-infrared femtosecond laser source are used to selectively polymerise photosensitive resins. The electronic excitation generated by the nearly simultaneous absorption of two photons is similar to that of a single photon with higher energy. This absorption provides a nonlinear energy distribution, centred at the laser’s focal point and with negligible absorption outside the immediate area of the laser’s focal volume (20). Upon absorption of this energy, photoinitiator molecules in the resin will begin the polymerisation process at locations known as “polymerisation voxels”, where the energy surpasses a certain threshold (19). In comparison with other techniques, 2PP shows improved geometry control, as well as scalable resolution, reducing equipment, facilities and maintenance costs commonly associated to etching and lithography-based methods. For this reason, researchers have exploited this technique to fabricate solid or hollow MN arrays using modified ceramics (21,22), inorganic-organic hybrid polymers (23,24), acrylate-based polymers (25,26), polyethylene glycol (27), and recently, water-soluble materials (28), with promising results.

In this work, we describe the fabrication of complex and highly detailed MN array master templates using 2PP 3D printing, in a simple and time-effective process which can prove beneficial in comparison with the established template manufacturing techniques previously described. We aimed at producing these templates in variable designs, to facilitate the comparison of different needle shapes, dimensions and geometries in the MN array performance. Using these templates, we reproducibly fabricated reusable MN array moulds, which were used to produce dissolving and hydrogel-forming MN arrays, with potential for use as drug delivery devices.

## Materials and Methods

### Materials

Poly (lactic acid) (PLA) was purchased from Ultimaker (Geldermalsen, Netherlands), and transparent LSR9–9508-30 silicone elastomer mix from Polymer Systems Technology (High Wycombe, UK). Gantrez® S-97, a copolymer of methyl vinyl ether and maleic acid (1,500,000 Da) and poly (vinylpyrrolidone) (PVP) (58,000 Da) were a kind donation from Ashland (Kidderminster, UK). Poly (vinyl alcohol) 9000–10,000 Da (PVA 10 K), poly (vinyl alcohol) 31,000–50,000 Da (PVA 50 K), poly (ethylene glycol) (PEG) 10,000 Da, anhydrous sodium carbonate (Na_2_CO_3_), anhydrous monobasic potassium phosphate, propylene glycol monomethyl ether acetate, isopropyl alcohol, trichloro(1H,1H,2H,2H-perfluorooctyl)silane and ibuprofen sodium (IBU Na) were obtained from Sigma-Aldrich (St. Louis, USA). Micronised cabotegravir sodium (CAB Na) was kindly supplied by ViiV Healthcare (Research Triangle (North Carolina), USA). Cryogel® SG/3 (gelatine) was acquired from PB Gelatins GmbH (Nienburg/Weser, Germany), Pearlitol 50 C (mannitol) from Roquette (Lestrem, France), phosphoric acid 85% from Amresco Inc. (Ohio, USA) and acetonitrile ≥99.9% from Honeywell Research Chemicals (Bucharest, Romania). Trifluoroacetic acid was purchased from Tokyo Chemical Industry (Tokyo, Japan). All other chemicals used were of analytical reagent grade.

### Template Design and Geometrical Optimisation

Master templates were designed and optimised with AutoCAD design software Fusion 360 (Autodesk, San Rafael, USA) and imported as a standard tessellation language (STL) file into a job preparation software (Describe, Nanoscribe, Germany). Describe then produces a general writing language (GWL) file for printing. The GWL code was modified in order to print large MN master templates (7 × 7 mm) within a reduced print time. The optimised parameters include writing field size, scaffold size, block size and print strategy.

### 3D Printing of Master Templates by Two-Photon Polymerisation

GWL files were imported to a Nanoscribe Photonic Professional GT 3D printer (Nanoscribe, Eggenstein-Leopoldshafe, Germany) for 2PP exposure. The machine is equipped with a laser source emitting 100 fs pulses at 80 MHz, with a wavelength of 780 nm. A droplet of IPS photoresin (Nanoscribe, Eggenstein-Leopoldshafe, Germany) was placed on top of a 25 × 25 mm coverslip which had been previously coated with a thin indium tin oxide (ITO) layer to produce a refractive-index mismatch. The laser beam was then focused within the resin using a 25x microscope objective (numerical aperture [NA] = 0.8). The designed structures were written layer-by-layer with ultraprecise piezo actuators, moving the sample in the axial direction after exposing each layer. In the lateral direction, the laser beam was guided by galvanometric mirrors parallel to the substrate. After exposure, the development process was performed in propylene glycol monomethyl ether acetate (PGMEA) for 30 min followed by 2 min rinsing in isopropyl alcohol (IPA) and subsequent blow-drying with nitrogen.

### Manufacturing of MN Array Moulds

The master templates were mounted on a PLA holder fabricated in-house using an Ultimaker 3 3D Printer (Geldermalsen, Netherlands), to allow the production of silicone micromoulds for MN array manufacturing. To prevent the adherence of the silicone elastomer to the master template during curing and de-moulding, the master templates were treated with trichloro (1H,1H,2H,2H-perfluorooctyl) silane. For this purpose, a weighing boat containing 10 μL of this substance and another one containing the master template were placed into a vacuum desiccator and kept under vacuum for 2 h. The MN array moulds were subsequently fabricated using the transparent LSR9–9508-30 silicone elastomer mix (part A: part B 1:1 *w*/w), according to the manufacturer’s instructions. The liquid mixture was degassed by centrifugation and poured into the holder containing the master template. The silicone filled template was then centrifuged at 3500 rpm for 15 min to remove any residual air bubbles and cured for 3–6 h at 60°C. The master template was allowed to cool down before removal of the silicone mould for MN array fabrication.

### Manufacturing and Characterisation of Dissolving and Hydrogel-Forming MN Arrays

Dissolving MN (DMN) arrays were initially prepared without any drug, from an optimised aqueous blend of 20% (*w*/w) PVP and 15% (w/w) PVA 50 K. An aliquot of approximately 200 mg of this polymeric blend was poured into each silicone mould and placed into a positive pressure chamber (Protima AT10 pressure tank; Richmond Scientific, Lancashire, UK) at 5 bar for 15 min. The MN arrays were then left to dry at room temperature for 24 h, followed by incubation at 37°C for 24 h (Genlab Ltd., Cheshire, UK). MN arrays were gently removed from the moulds, sidewalls were removed using a pre-heated disposable scalpel and then stored in a desiccator until further use.

For the preparation of the so-called ‘super-swelling’ hydrogel formulation (29), a stock solution of the copolymer Gantrez® S-97 (40% *w*/w) was initially prepared using deionised water. The formulation was subsequently prepared by combining Gantrez® S-97 (20% w/w), PEG 10,000 Da (7.5% w/w), Na_2_CO_3_ (3% w/w; modifying agent) and deionised water. This aqueous blend was centrifuged at 3500 rpm for 15 min to remove any air bubbles and then approximately 0.8 g of the formulation was slowly poured into MN array moulds. Cast moulds were placed into a positive pressure chamber as previously described to facilitate filling of the needle tips. After 15 min, the moulds were dried at room temperature for 48 h and then incubated at 80°C for 24 h to induce chemical cross-linking between Gantrez® S-97 and PEG 10,000 Da by esterification. The MN arrays were carefully separated from their moulds and the sidewalls removed using a hot scalpel. MN arrays were visually inspected using either a Leica EZ4 D digital light microscope (Leica Microsystems, Milton Keynes, UK) or a Tabletop TM 3030 scanning electron microscope (Hitachi, Tokyo, Japan).

#### Insertion Efficiency

To evaluate the insertion properties of the various MN array designs, a previously validated skin model composed of stacked layers of Parafilm M® (Bemis Company Inc., Soignies, Belgium) was used (30). Briefly, the texture analyser was set on compression mode and the different MN arrays were attached to the cylindrical probe using double-sided adhesive tape. Subsequently, the probe was lowered onto the skin model at a speed of 0.5 mm/s until the required force was exerted and held for 30 s. Once the target force was reached, the probe was moved upwards at a speed of 0.5 mm/s. Different forces were used depending on the MN formulation, namely 0.082–0.246 N/needle for DMN arrays and 0.4–2.13 N/needle for HFMN arrays. After insertion, the MN arrays were removed from the skin model and the Parafilm M® layers were separated to count the individual holes created by the MN array in each layer using light microscope imaging. To facilitate visualisation, each Parafilm M® layer was placed between two polarising filters.

Optical coherence tomography (OCT) was also used to visualise, using an EX1301 OCT Microscope (Michelson Diagnostics Ltd., Kent, UK), the insertion of HFMN arrays in neonatal full thickness porcine skin, collected from still-born piglets within 24 h from birth. Full-thickness skin was excised using a disposable scalpel, wrapped in aluminium foil and stored at −20°C until use. Before use, the skin was thawed in phosphate-buffered saline (PBS, pH 7.4) at room temperature for 30 min and carefully shaved using a disposable razor. HFMN arrays were inserted manually into the skin, holding thumb pressure for 30 s, and subsequently imaged by OCT.

#### Drug Loading in DMN Arrays

DMN arrays containing CAB Na were prepared using an aqueous blend of 20% (*w*/w) PVA 10 K and 20% (w/w) PVP mixed at (1:1 w/w), to which 60% w/w CAB Na (calculated as CAB base) was added. The first layer of the DMN arrays was prepared by dispensing 100 mg of the drug-loaded polymeric blend into each silicone mould and placing these into a positive pressure chamber at 5 bar for 3 min. The excess of drug-loaded polymeric blend was carefully removed from the surface and the moulds were placed again into the positive pressure chamber, with pressure applied for 30 min at 5 bar. This first layer of the DMN arrays was subsequently dried overnight at room temperature. The second layer was then prepared by pouring the previously described drug-free polymeric blend on top of the first layer under vacuum and placing the moulds into the positive pressure chamber at 5 bar for 15 min. Finally, the DMN arrays were dried at room temperature for 24 h and at 37°C for 24 h, as previously described. Dried DMN arrays were gently removed from the moulds, sidewalls were removed using a pre-heated disposable scalpel and the arrays were stored in a desiccator until further use.

The drug content of each DMN array was determined by dissolution (*n* = 3 of each design) in 5 ml of deionised water. After magnetic stirring at 600 rpm for 30 min at room temperature, ensuring complete dissolution of the DMN array, an aliquot of 100 μL of the resultant drug suspension was collected and diluted with 900 μL of acetonitrile and vortexed for 30 s to completely dissolve the drug for quantification. This solution was centrifuged at 14800 rpm for 10 min and an aliquot of 300 μL of the clear supernatant solution was collected for analysis with a validated reversed-phase high performance liquid chromatography (RP-HPLC) method with ultraviolet (UV) detection, as described in section 2.6.

#### Ex Vivo Drug Deposition in Porcine Skin Using DMN Arrays

Drug deposition in skin was performed using neonatal full-thickness porcine skin, obtained from still-born piglets as described in section 2.5.1. Before performing the drug deposition studies, skin samples thawed in PBS (pH 7.4) and shaved were placed on top of PBS-soaked absorbent paper to provide support and hydration of the dermis side of the skin during the experiment. The skin surface was dried with tissue paper and drug-loaded DMN arrays (*n* ≥ 3 from each design) were inserted into the skin using manual force for 30 s. A circular stainless-steel cylinder block of 13.0 g was placed on top of the MN arrays to keep them in place. Following this, the skin with the inserted DMN arrays was incubated at 32 ± 1°C for 24 h, after which the remainder of the drug-loaded arrays was gently removed. The amount of drug deposited on the skin surface was washed by applying 1 mL of PBS (pH 7.4) and carefully wiping the skin surface with tissue paper. Skin sections at the DMN array application site were collected using a disposable scalpel, cut into small pieces using scissors and placed into a 2 mL Eppendorf tube for storage at −20°C until analysis.

To quantify the amount of CAB Na present in skin samples, two stainless-steel beads and 500 μL of deionised water were added to each sample, followed by homogenisation for 15 min using a Tissuelyser LT (QIAGEN, Manchester, UK) to solubilise the remaining DMN arrays deposited in the skin. Subsequently, 1 mL of acetonitrile was added to each sample and the mixture was homogenised for another 15 min to solubilise the drug. Each sample was then diluted with 3.5 mL of acetonitrile: water (1:1 *v*/v) and vortexed for 5 min. An aliquot of 100 μL of this final skin homogenate was diluted with 900 μL of acetonitrile, vortexed for 30 s and centrifuged at 14800 rpm for 10 min. The clear supernatant solution was collected and analysed for CAB Na content using the RP-HPLC-UV method described in section 2.6.

#### In Vitro Drug Permeation Using HFMN Arrays

For the in vitro permeation of IBU Na using HFMN arrays, lyophilised wafer-like reservoirs of this drug were prepared as previously described (29). For this purpose, a mixture of gelatine (10% *w*/w), mannitol (3% w/w) and IBU Na (40% w/w) in deionised water was prepared in a DAC 150 FVZ-K SpeedMixer™ (Synergy Devices Ltd., High Wycombe, UK) at 3000 rpm for 60 s and sonicated at 37°C for 60 min. Approximately 250 mg of this formulation was then cast into open-ended cylindrical moulds (diameter 13 mm, depth 3 mm) and placed into a Virtis Advantage® Bench-top Freeze Drier System (SP Scientific, Warminster, USA) for freeze-drying according to a previously reported protocol (31). After visual inspection, the lyophilised wafer-like reservoirs were stored at 2–8°C until further use.

Modified Franz diffusion cells were used in the permeation assay, for which dermatomed (350 μm), neonatal porcine skin obtained from stillborn piglets was shaved and attached to the donor compartment of the diffusion cells. HFMN arrays were then inserted into the skin by applying manual pressure for 30 s. A 20 μl aliquot of PBS solution (pH 7.4) was subsequently pipetted onto the centre of the HFMN array to promote adhesion of the lyophilised wafer-like reservoir containing approximately 100 mg IBU Na. A stainless steel weight (11.0 g) was placed on top of the lyophilised wafer-like reservoir to prevent expulsion of the HFMN array from the skin upon swelling. The acceptor compartment contained PBS solution (pH 7.4) which was thermostatically maintained at 37°C ± 1°C. The donor compartment of the apparatus was clamped onto the receiver compartment, and the donor compartment and sampling arm were sealed using Parafilm M®. A 0.2 mL sample of the acceptor compartment solution was removed from each Franz cell at predefined time points using a 1 ml syringe with an 8 cm needle. The sample volume was replaced with an equal volume of PBS solution (pH 7.4). The sample was subsequently centrifuged at 13,400 rpm for 15 min before quantification using a previously validated RP-HPLC-UV method, described in section 2.6.

### HPLC Method Development and Validation

Two RP-HPLC methods with UV detection were developed and validated in this study, for quantification of CAB Na and IBU Na. In both, the analysis was performed on an Agilent 1220 Infinity LC system (Agilent Technologies UK Ltd., Stockport, UK) and Chemstation® B.02.01 software (Agilent Technologies UK Ltd., Stockport, UK) was used for chromatogram analysis.

The analysis of CAB Na was performed with an Inertsil® ODS-3 C18 column (5 μm particle size, 150 mm length, 4.6 mm internal diameter) (GL Sciences Inc., Tokyo, Japan) and detection at 257 nm. The column temperature was maintained at 40°C. Separation was performed using an isocratic method, with a mobile phase composed of acetonitrile and water (containing 0.1% *w*/w trifluoroacetic acid, pH 2.35) (70:30, *v*/v), which was pre-filtered through a 0.2 μm pore size filter (Alltech, Deerfield, IL), and delivered at a flow rate of 0.8 mL/min for 8 min. The injection volume was 40 μl. Standard CAB Na samples (10.5–105 μg/mL) were prepared in a mixture of acetonitrile and water (50:50, *v*/v).

In the case of IBU Na, the column used was an Agilent Zobrax Eclipse XDB-C18 (5 μm particle size, 150 mm length, 4.6 mm internal diameter) kept at a temperature of 20°C. The mobile phase was composed of acetonitrile and an aqueous solution of potassium phosphate at 0.02 M (pH 2.8) (70:30 v/v) and UV detection was set at 220 nm. The flow rate was 1 mL/min for a run time of 10 min and the injection volume was 50 μL. Standard samples of IBU (0–08 – 50 μg/ml) were prepared in PBS (pH 7.4).

Both methods were validated according to the International Conference on Harmonisation (ICH) guidelines (32). The parameters assessed during method validation were specificity, linearity, range, accuracy, precision, limit of detection (LoD), and limit of quantification (LoQ). All the calibration plots were subsequently collated to generate one representative calibration curve. Least squares linear regression analysis and correlation analysis were performed. The LoD and LoQ were determined using the standard deviation (S.D.) of the response and slope of the calibration curve, as described in the ICH guidelines.

### Statistical Analysis

All data are expressed as means ± SD of at least three replicates. Least squares linear regression analysis, correlation analysis, LoD and LoQ were performed using Microsoft® Office 365 ProPlus Excel (Microsoft Corporation, Redmond, USA). Statistical analysis was performed using GraphPad Prism® 7 (GraphPad Software, San Diego, USA), using the Kruskal-Wallis test with Dunn’s multiple comparison test.

## Results

### Design, Optimisation and Manufacturing of Master Templates by 2PP 3D Printing

**Fig. 1:**
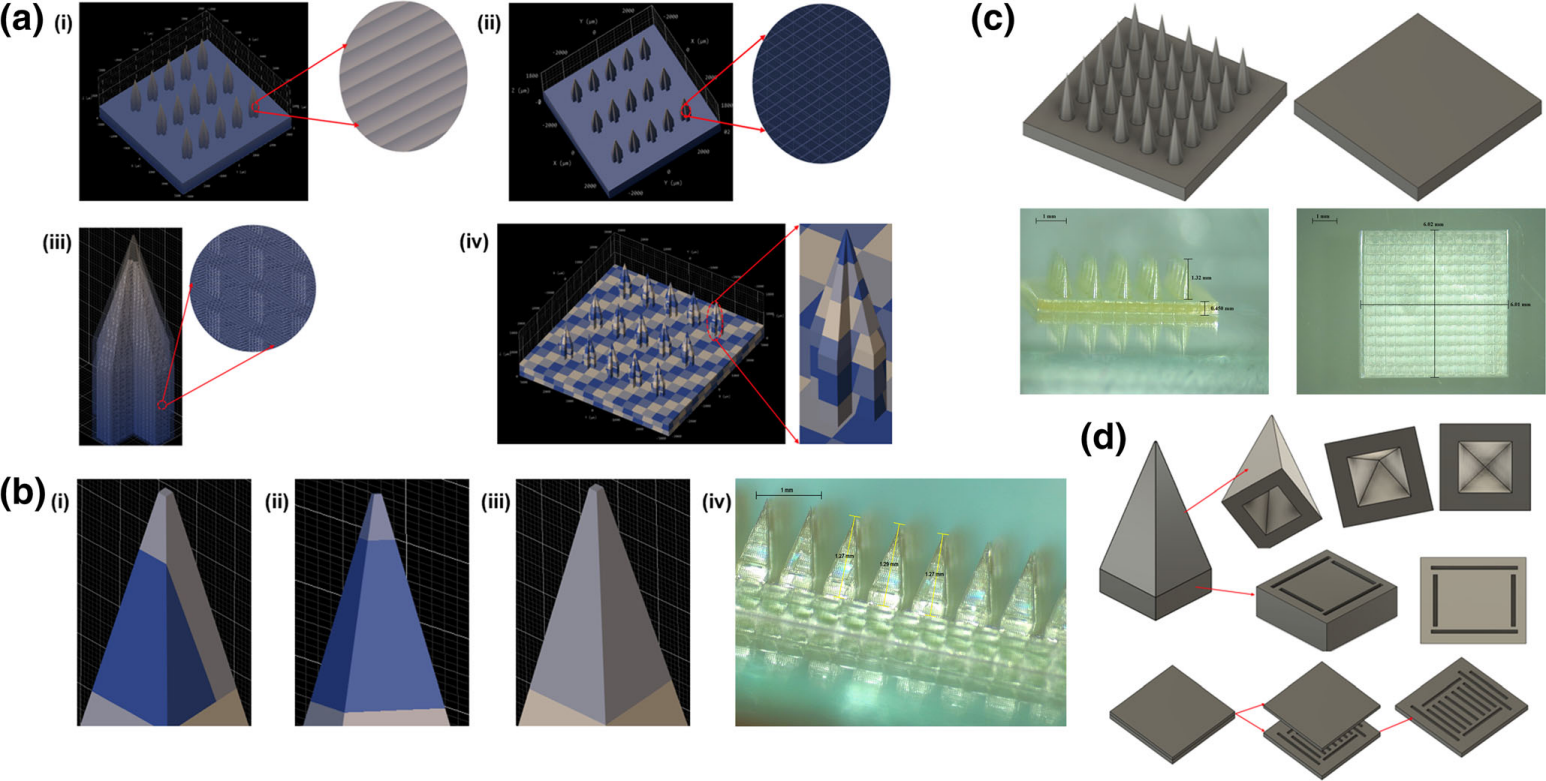
Optimisation of different 3D printing parameters (Describe software settings): (**a**) (**i**) distance between layers (*“slicing”*); (**ii**) shell parameters (distance between hatching lines within a layer – *“hatching distance”*, shell contour count, and number of filled slices at the bottom of the shell – *“base slice count”*); (**iii**) scaffold parameters (spacing between scaffold walls and floors, thickness of scaffold walls and scaffold floor); and (**iv**) “splitting” mode (block width in X and Y direction, block height and block offset in X, Y and Z direction); (**b**) effect of block size and position in printing the needle tip with (**i**) two blocks, (**ii**) one short block in Z direction, and (**iii**) one single and large block [(**iv**), printed example]; (**c**) Two-step 3D printing of long MN master templates (1.3 mm high needles) and additional baseplate; (**d**) cavities introduced in the MN, pedestal and baseplate designs.

To ensure accurate and reproducible printed structures, 2PP requires optimisation of key parameters including writing field size, scaffold size, block size, and print strategy (Fig. 1). The influence of these parameters on print time, mechanical stability, resolution, and quality of the master templates was evaluated in this study.

The use of “shell & scaffold filling”, recommended for printing large structures, allows a 2.7-fold reduction in print time in comparison with printing in solid mode, for a 0.5 mm high 3 × 3 mm square, from 43 to 16 h (data not shown). Adjusting printing parameters such as hatching density, slicing distance, and space between the horizontal and vertical scaffold walls may lead to a further 2.5-fold reduction in print time. However, an inappropriate setting of these parameters (e.g. slicing and hatching distance above 1.7 μm, large spacing between the horizontal and vertical scaffold walls (above 35 μm) and a small wall width below 3 μm) can lead to mechanical instability (curvature/bending) of the printed structure after development.

The printing process is performed block by block in the X and Y directions. The block size can affect the printing time and quality of the printed structure, since smaller block sizes lead to a higher total number of blocks and therefore a higher printing time, affecting print resolution. For example, increasing the block size from 100 to 400 μm decreases printing time by half. Additionally, block size can also affect roughness, due to the stitching effect between blocks. The block position is also an important factor affecting the quality of the printed needle, particularly in terms of the tip sharpness, since dividing the needle tip area in two or more blocks can affect this feature (Fig. 1b**(i)**). Another critical parameter is the block height in the Z direction, since reducing the block height below 20–30 μm may cause print distortions during the stitching step and lead to tip breakage when manufacturing the MN array moulds (Fig. 1b**(ii)**). Finally, printing the upper part of the needle as a single and large block was required to achieve sharpness and robustness.

Given the limited working space available between the sample holder and the moving stage of the microscope, the height of the printed structures was also limited. To overcome this issue and enable the printing of MN master templates with 1.3 mm in height, the structure was printed in two steps, namely a 0.5 mm high baseplate and a top structure containing the 1.3 mm high MN on another 0.5 mm high base (Fig. 1c). The two components were then aligned and glued in the custom-made PLA holder described in section 3.2.

In this 3D printing technique, long printing times can lead to the resin material spreading to the bottom side of the objective and consequently to an incorrect printing of the final structure. By reducing unnecessary features of the structure (e.g. a very large baseplate) and including cavities in both the needle and the baseplate designs (Fig. 1d), the total volume of the design was reduced by approximately 7% in comparison with the original unmodified structure, which also led to a reduction in printing time. However, the cavity volume and position must be carefully designed to avoid mechanical instability of the final structure. We observed that the cavity in the upper part of the needle should be in its centre and with a maximum volume of around 20% of the total upper part volume. Additionally, the sidewall of the needle should be at least 100 μm thick to maintain mechanical stability. For the designs with a pedestal under the needle tip, a discontinued set of trenches (30 μm in width and 50 μm in depth) at the edge of the structure were found to be optimal to avoid any continuous connection between the cavities in the upper and lower part of the needle. The same strategy was applied for the baseplate of the master template, with a discontinued set of trenches (200 μm in width and 100 μm in depth) being distributed in the centre of the structure. Finally, our results indicated it was important to avoid the use of air pressure or a nitrogen gun to dry the printed structure directly after rinsing with IPA, as it led to bending of the needle tips.

**Fig. 2:**
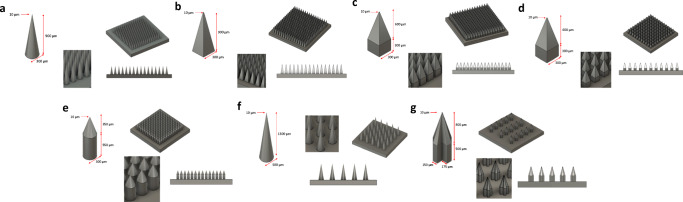
Final CAD-assisted image of all MN array designs developed with the dimensions of individual needles, (**a**) D1 to (**g**) D7.

Figure 2 and Table I summarise the physical dimensions and characteristics of the seven MN array designs developed and manufactured by 2PP 3D printing in an average time of 43 h, using the optimised printing technique previously described.

**Table I:** Summary of the Physical Dimensions and Theoretical Needle Volume (in Dry State) of All Microneedle (MN) Array Designs Evaluated in this Study

	Number of needles	MN shape	Height (μm)	Base width / side (μm)	Interspacing (μm)	Theoretical needle volume/array (mm^3^)
D1	256 (16 × 16)	Full conical	900	300	100	5.38
D2	256 (16 × 16)	Full pyramidal	900	300	100	6.91
D3	256 (16 × 16)	Cuboidal base / pyramidal tips	900 (300 + 600)	300	100	11.52
D4	121 (11 × 11)	Cuboidal base / pyramidal tips	900 (300 + 600)	300	300	5.45
D5	196 (14 × 14)	Cylindrical base / conical tips	900 (550 + 350)	300	100	9.26
D6	25 (5 × 5)	Full conical	1300	500	500	2.13
D7	15 (5 × 3)	Cross-shaped	1300 (500 + 800)	500 (175 + 150 + 175)	500	1.49

### Assembling of Master Templates and Manufacturing of MN Array Moulds

**Fig. 3:**
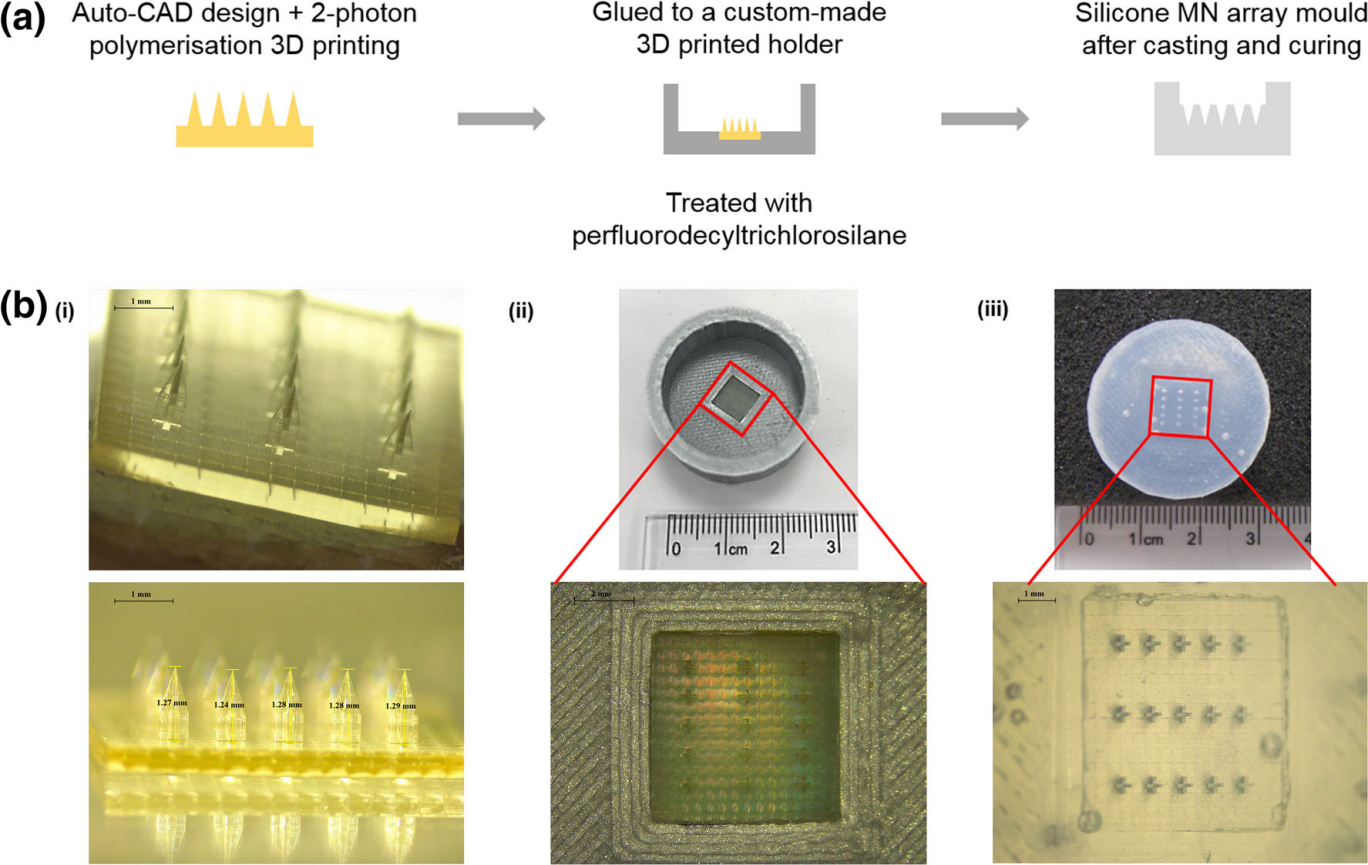
(**a**) Schematic representation of the method used to assemble master templates produced by 2PP 3D printing to fabricate silicone MN array moulds. (**b**) Representative light microscope images of the different steps of the manufacturing process (D7 design shown as an example): (**i**) master template, (**ii**) master template mounted on PLA holder and (**iii**) silicone MN array mould.

To create a housing for silicone MN moulds, 3D printed master templates were mounted on a PLA holder, designed and 3D printed in-house. In the silicone curing process, as we observed that the PLA holder shrunk during the first heating cycle (≥ 80°C), the protocol was optimised by reducing the curing temperature to 60°C. Initial trials of MN mould fabrication led to breakage of the master template needles and difficulties to remove the MN array moulds from the templates. This was attributed to the interaction between the surface of the UV-cured resin master templates and the silicone elastomers. To avoid this interaction, the protocol was optimised to include a previous treatment of the master templates with silane. Other parameters such as the duration of the silane treatment, air removal from the liquid silicone elastomer mixture by centrifugation and duration of silicone curing were also optimised to achieve an optimal and time-efficient manufacturing process. The final protocol, described in section 2.4, allowed for the manufacturing of up to 40 MN moulds without any damage to the master templates. Figure 3 summarises the moulding protocol and provides images of the different steps of the process.

### Pharmaceutical Analysis

To allow the quantification of the model drugs used in further studies with the developed MN arrays, two RP-HPLC methods with UV detection were developed and validated according to ICH guidelines. The validation parameters for both drugs are summarised in Table II, and exemplar calibration curves for each drug are shown in Fig. S2.

**Table II:** Summary of the RP-HPLC-UV Validation Parameters Obtained in the Quantification of CAB Na and IBU Na in PBS (pH 7.4)

	Range (μg/mL)	Slope	y-intercept	r^2^	LOD (μg/mL)	LOQ (μg/mL)
CAB Na	5.27–105.43	131.50	−377.28	0.9981	0.7	1.9
IBU Na	0.08–50	90.92	3.44	1.0000	0.07	0.22

### Application of the Technology to Dissolving Formulations

#### Fabrication and Characterisation of DMN Arrays

**Fig. 4:**
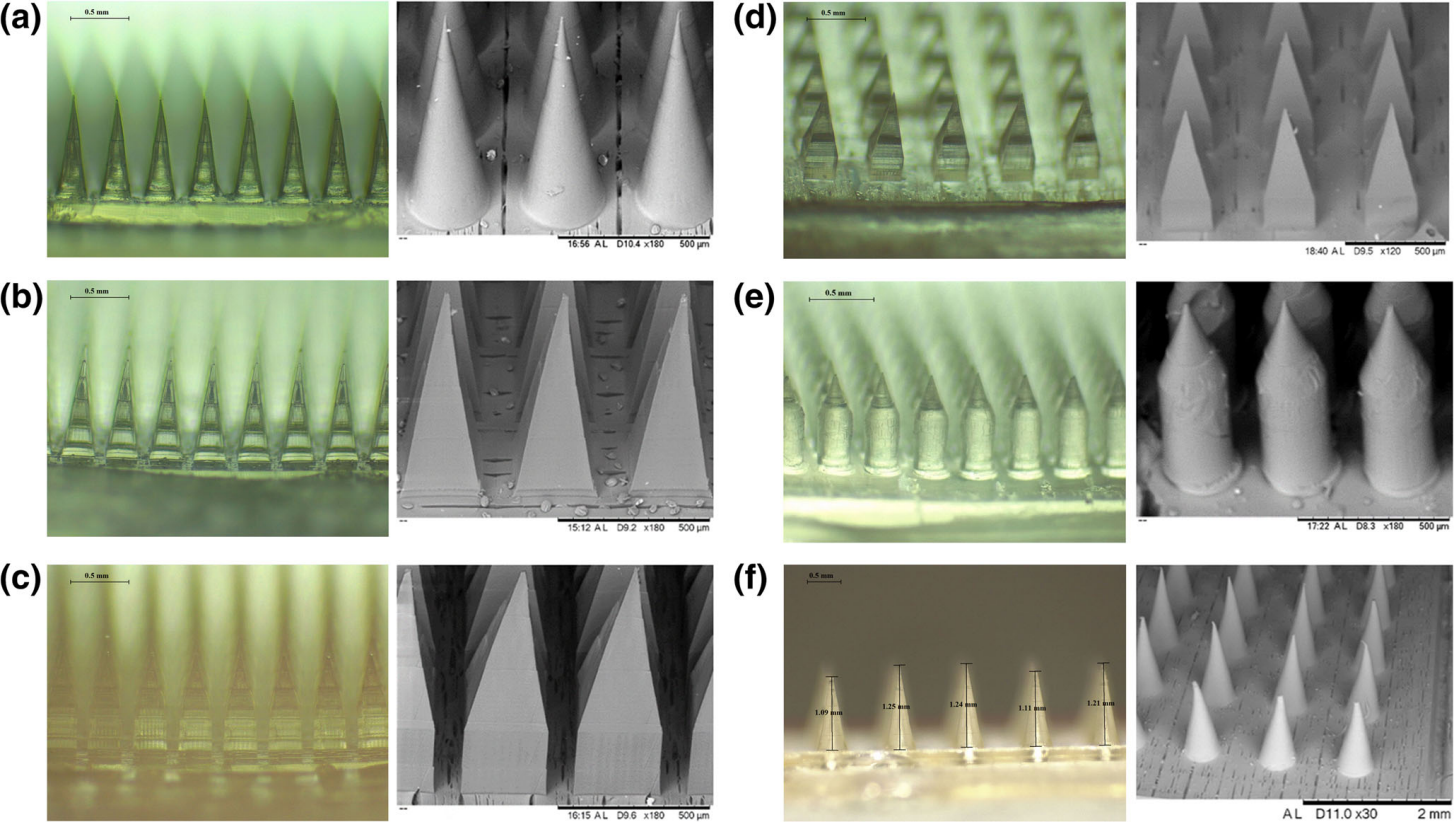
Light microscope (left) and scanning electron microscope (right) images of the seven MN array designs prepared with dissolving formulation (**a**, D1 to **f**, D6).

DMN arrays were prepared as described in section 2.5 with satisfactory mechanical properties. As shown in Fig. 4, the base width and interspacing of the MN array obtained were identical to that of the master templates. However, the needle height was consistently reduced by 5–12% in comparison with the values defined in the templates, possibly due to formulation shrinkage upon drying.

The various DMN array designs were characterised for their insertion capability in the validated skin-simulant membrane consisting of 10 layers of Parafilm M® (30). Since the insertion force might vary from one person to another, these studies were performed at low (minimum force required to insert MN into the skin), intermediate and high insertion forces (0.082, 0.164 and 0.246 N/needle, respectively).

**Fig. 5:**
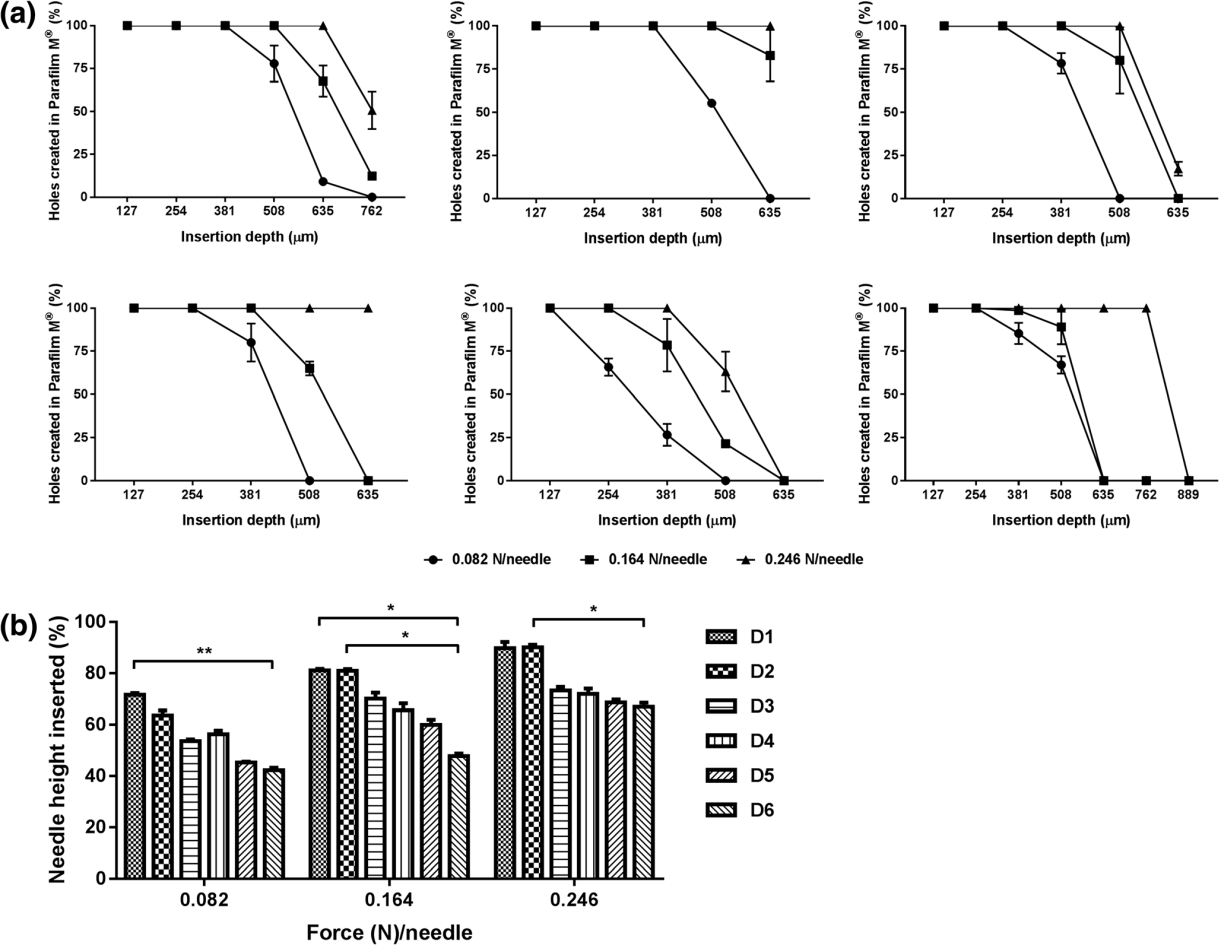
Insertion of the different DMN arrays into Parafilm M®. (**a**) Percentage of holes created by each MN array in Parafilm M® layers (each 127 μm in height) in relation with the total number of MN per array. Upon application of different forces, as measured by (**a**) light microscope and (**b**) OCT images. All results shown as means ± SD, *n* = 3.

All DMN arrays were inserted into Parafilm M® when a low force was applied, as seen in Fig. 5. However, the insertion depth profiles varied depending on the design and the applied insertion force, with none of the MN arrays showing complete insertion. D1 and D2 exhibited the highest insertion depth values, with 72–90% and 64–90% of their needle height being inserted, respectively. This suggested that D1 (full conical shape needles) has slightly better insertion capabilities than the other designs at low insertion forces. However, no significant differences were observed at the highest insertion force tested (*p* > 0.09). In the case of D3 and D4, their percentage of insertion depth at low force was of 54–56% of the needle height, corresponding to 76–80% of pyramidal tips length. Maximum insertion depth of these designs was achieved with an intermediate insertion force, reaching approximately 70% of the needle height (approximately 97% of the pyramidal tips length). Increasing the insertion force resulted in a further increase in the insertion depth, though no significant differences were observed (*p* > 0.05). D5 and D6 exhibited similar insertion profiles to that obtained with D1 MN array design. However, the insertion efficiency of D6 was significantly lower than that of D1 or D2 at all applied forces (Fig. 5b), ranging between 42 and 67% of the needle height.

#### Drug Loading in Different DMN Array Designs

**Fig. 6:**
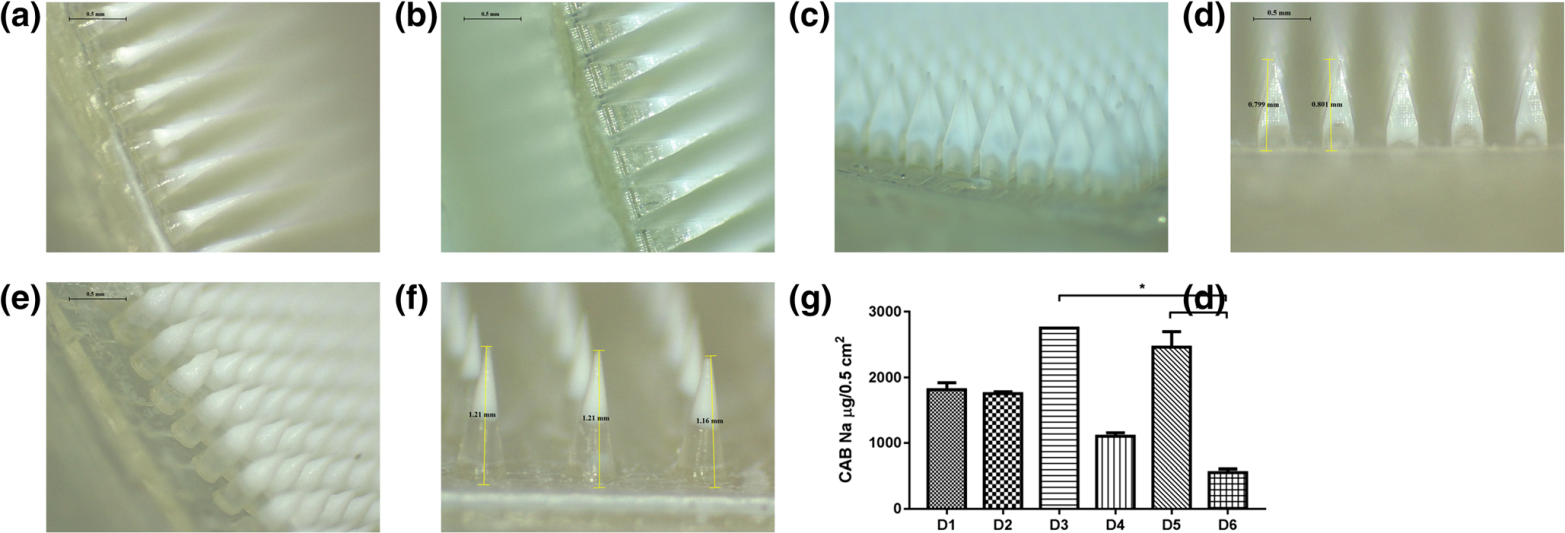
Light microscope images of CAB Na-loaded DMN arrays (**a**, D1 to **f**, D6). (**g**) CAB Na content per array area in each of the developed DMN array designs (means ± SD, *n* ≥ 3).

To assess the potential of the different DMN array designs for transdermal drug delivery of a hydrophobic molecule, micronised CAB Na was used as model drug. This drug is available as a microcrystalline powder which is sparingly water soluble (0.414 μg/ml). To reduce drug wastage, a two-step casting method was developed, allowing the concentration of the drug only within the needle tips. To maximise the drug amount loaded per array, the first layer of the DMN arrays was cast using a formulation containing 60% (*w*/w) CAB Na, calculated based on the total solid content of the formulation. As can be seen in Fig. 6, the bilayer DMN arrays were efficiently formed using all designs. The effective drug content in each DMN array ranged between 0.533 and 2.891 mg, depending on the geometry and the number of needles per array in each design. D3 and D5 exhibited the highest drug loading (2.75 and 2.46 mg/array, respectively), while D4 and D6 showed the lowest values (1.1 and 0.55 mg/array, respectively), due to the lower number of needles in these MN array designs. D1 and D2 showed intermediate drug loading values (1.81 and 1.76 mg/array, respectively), correlating with the total volume of each DMN array design.

#### Ex Vivo Drug Deposition in Porcine Skin

**Fig. 7:**
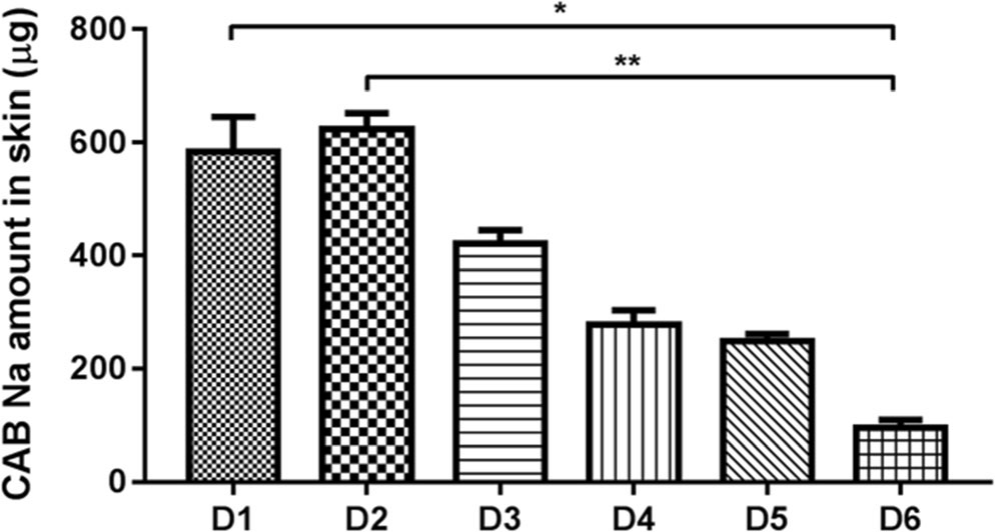
Amount of CAB Na (in μg/0.5 cm^2^) deposited in neonatal porcine skin following DMN array insertion (means ± SD, *n* ≥ 3).

Drug-loaded DMN arrays were efficiently inserted in neonatal porcine skin, as evidenced in Fig. S1. The amount of CAB Na deposited in porcine skin ranged between 95.1 and 624.5 μg, as can be seen in Fig. 7. These values varied with the DMN array design, with D2 showing the highest amount of drug deposition and D6 exhibiting the lowest. This was in agreement with the previously described insertion results.

### Application of the Technology to Hydrogel-Forming Formulations

#### Manufacturing and Characterisation of HFMN Arrays

**Fig. 8:**
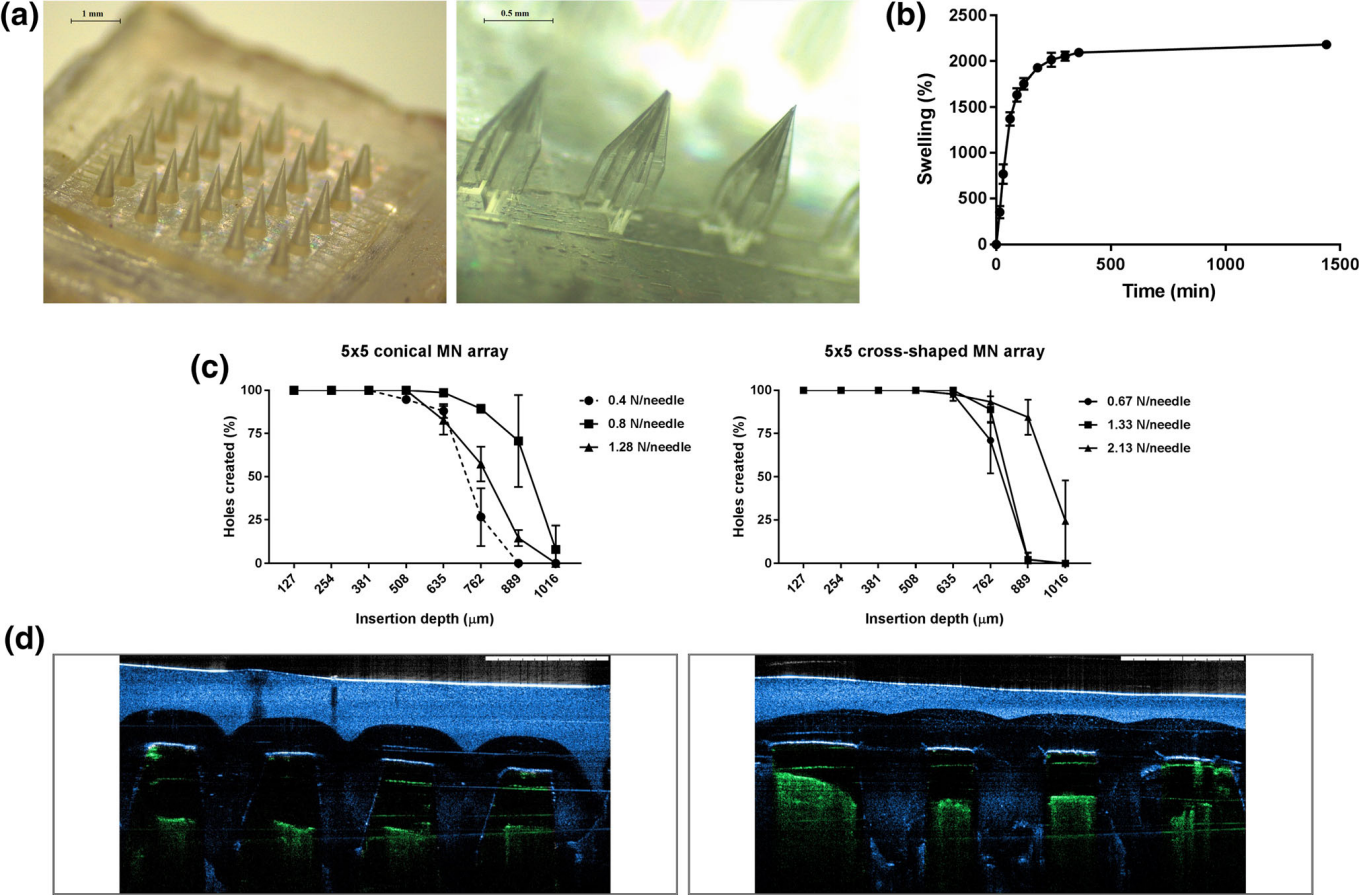
(**a**) Light microscope images of the two different HFMN array designs (D6, left; D7, right). (**b**) Swelling profile of the hydrogel-forming formulation. (**c**) Insertion of HFMN arrays into Parafilm M®, upon application of different forces. (**d**) Representative optical coherence tomography (OCT) images of the insertion of HFMN arrays into full thickness porcine skin. Images artificially coloured to facilitate visualisation (skin is seen in green and HFMN arrays in blue). All results shown as means ± SD, *n* ≥ 3.

HFMN arrays were prepared using two different designs, one with 5 × 5 conical needles (D6–0.5 cm^2^ array area, 1.2 mm needle height, 500 μm base width and 1 mm tip-to-tip needle spacing) and the other with 5 × 3 cross-shaped needles (D7–0.5 cm^2^ array area, 1.3 mm [500 + 800 μm] needle height, 150 μm lateral width and 1 mm tip-to-tip needle spacing) (Fig. 8a). To assess the utility of these MN arrays for transdermal application, they were characterised in terms of their swelling and insertion into skin models.

HFMN arrays showed fast swelling upon immersion in PBS (pH 7.4), with the weight of the formulation increasing to 1371 ± 75% of the initial value in the first hour, and stabilising at 2183 ± 48% after 24 h of incubation (Fig. 8b). Both types of HFMN arrays could also be inserted into Parafilm M® to a depth of 889–1016 μm (7–8 Parafilm M® layers), depending on the force applied (Fig. 8d). This insertion study showed some differences in the insertion of the two MN array designs, with D6 being more efficiently inserted when a force of 0.8 N/needle was applied and D7 requiring the application of 2.13 N/needle to achieve the same insertion efficiency. Manual insertion of these MN arrays into full thickness porcine skin was also assessed by OCT imaging (Fig. 8e).

#### In Vitro Drug Permeation with HFMN Arrays

**Fig. 9:**
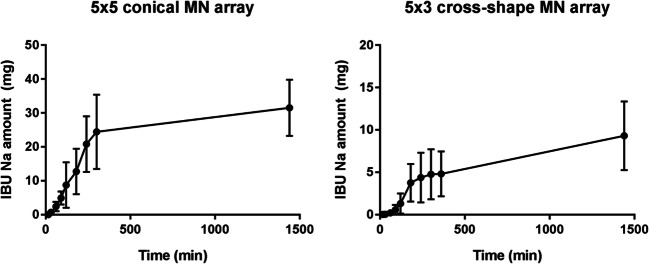
In vitro permeation of IBU Na across dermatomed porcine skin (350 μm in thickness) using 5 × 5 conical and 5 × 3 cross-shaped MN arrays and a lyophilised wafer-like reservoir (means ± SD, *n* ≥ 3).

The potential of the developed HFMN arrays to be used as transdermal drug delivery systems for low potency drugs was evaluated using IBU Na as a model drug. As shown in Fig. 9, both types of HFMN arrays allowed a controlled permeation of IBU Na across dermatomed skin in a period of up to 24 h, though in different amounts. The D6 HFMN arrays led to a higher total amount of IBU Na delivery, reaching approximately 32 ± 8% of the initial drug content of the lyophilised wafer-like reservoir (100 mg). On the other hand, in the case of the D7 HFMN arrays, the drug amount measured in the acceptor compartment of the diffusion cells at 24 h post-insertion only reached approximately 9 ± 4% of the initial value.

## Discussion

MN array fabrication requires the development of geometrically complex and precisely controlled topographies, capable of carrying large payloads of therapeutic drugs. Two-photon polymerisation 3D printing requires optimisation of key parameters to ensure the required accuracy and reproducibility of MN features can be delivered over a large area within a short print time. This has been previously described by Ströer *et al.* (33), who studied the influence of printing process parameters on the microgeometry and surface roughness of 3D printed microstructures as standards for optical instrument calibration. Our optimisation process, through the modification of the MN array design and the two-step print strategy described in section 3.1, allowed us to overcome the inherent limitations of 2PP 3D printing for this application. The position, dimensions and volume of the cavities created within the MN array design played a crucial role to reduce printing time and ensure good print quality and excellent mechanical stability after development. These results therefore validate the optimisation process to print complex MN arrays.

One of the most relevant advantages of MN arrays is their versatility to be applied in the delivery of different types of drugs, from small molecules to more complex biomacromolecules such as proteins, peptides and antibodies (1,2). In this work, we particularly focused on polymeric MN arrays prepared in either dissolving or hydrogel-forming formulations. DMN arrays have attracted attention mainly due to their demonstrated ability to efficiently deliver hydrophobic and hydrophilic drugs, vaccines and nanoparticles across the skin (34–36). On the other hand, HFMN arrays have shown their potential to be used, not only in the delivery of various drug molecules, but also in the extraction of skin interstitial fluid for diagnostic purposes (29,37).

The ability of MN arrays to efficiently deliver drugs is considerably controlled by their physical and mechanical properties, including needle strength, insertion depth and drug loading capacity (1,38). These properties are affected not only by the materials used in the fabrication of the MN arrays but also in the design of the array, including key parameters such as needle height, base width, aspect ratio, interspacing and shape. In the literature, the most common shape used for MN array design is either conical or pyramidal, with needle heights between 50 and 900 μm, base width ranging from 50 to 500 μm and an interspacing of 50–600 μm (2,39). A careful selection of these parameters is essential to produce functional MN arrays with the highest possible drug loading, maximum insertion capacity and acceptable mechanical strength, potentiating drug delivery efficiency.

Herein, we investigated the potential of using 2PP 3D printing technology to create a plethora of MN array designs that could enhance dermal and transdermal drug delivery. A total of eight designs were created using this technology, and fabricated in a time-effective manner to produce master templates used in the manufacturing of MN array moulds. Across all designs, the tip radius of the MN was kept constant at 10 μm, as this parameter has been shown to be critical for skin insertion (40). Designs D1 and D2 were developed from the previously mentioned conical and pyramidal shapes (30,41), increasing needle height and number of needles per array to potentially improve drug loading and MN array insertion. These designs allow a high number of needles in a small array (0.5 cm^2^), therefore having a high total theoretical volume per array to potentially load higher drug amounts. To assess the effect of this parameter, D3 design was created by adding a cuboidal base to the pyramidal tips, while maintaining the overall needle height and base width. This allowed for an increase of 1.7-fold in the theoretical needle volume (6.91 to 11.52 mm^3^). Similarly, D5 design was developed from D1 by adding a cylindrical base to the conical tips and therefore increasing the theoretical needle volume by the same factor (5.38 to 9.26 mm^3^). To evaluate the effect of needle interspacing, D4 design was produced with similar characteristics to D3, though having higher interspacing and therefore a smaller number of needles per array. Consequently, this MN array also has a theoretically smaller total needle volume in comparison with D3 (11.52 vs 5.45 mm^3^). Finally, longer needles were included in designs D6 and D7, as they can be useful for particular applications such as the treatment of skin cancer lesions, in which highly localised drug delivery into deeper areas of the lesion can be beneficial. In fact, research has shown how increasing needle height directly relates with increasing drug delivery efficiency (Yan *et al.*, 2010). The versatility and high level of resolution of the 2PP technique allowed us to produce the innovative cross-shaped MN design (D7), which maximises the surface area of the needles, and could potentially encourage an increased absorption of skin interstitial fluid for diagnostic purposes.

The needles obtained in this study showed high level of detail, sharpness and smooth edges, which was expected to contribute to their efficient insertion in skin models. In our study, conical shaped DMN arrays (D1) showed better insertion capabilities at low force than other alternative shapes with the same dimensions, though no significant differences were observed at the highest force tested (*p* > 0.99). Increasing the height of these needles (D6 vs. D1) led to efficient insertion yet requiring the application of higher forces. It was also possible to observe that adding a cuboidal or cylindrical base to the needles did not significantly change the insertion efficiency of those MN arrays (D1 vs. D5, *p* ≥ 0.09 and D2 vs. D3, p > 0.99). Due to their triangular cross-section, conical and pyramidal designs receive the applied force in a uniform distribution, focused on the tip of the needle, which may facilitate their insertion into the skin. On the other hand, designs with a cuboidal or cylindrical base showed a different distribution of the compression force applied increasing the resistance of the skin to their insertion. A similar result was observed when comparing conical and cross-shaped MN array designs (D6 vs. D7) with the hydrogel-forming formulation. Here, alongside the force distribution discussed above, the small angle between the edge of the conical needle and its central axis (10.9°), in comparison with that of the cross-shaped needle (18.1°), may explain the insertion results obtained with these prototypes. As observed by Bal *et al.* (42), the shape and corresponding needle sharpness strongly influences the size of the conduits created in the skin after application. Sharper needles, such as those with conical and pyramidal shapes, require lower insertion forces and lead to different penetration depths of the loaded drug molecule in the skin.

Another parameter we evaluated in this study was the spacing between the needles, comparing designs D3 and D4. We observed that increasing this parameter was slightly beneficial for insertion at low forces, as seen by the results obtained with D4. However, no significant differences were observed between these prototypes in terms of insertion depth at any tested force (*p* > 0.99). A similar result was also observed when comparing the insertion results of D1 and D6 with the dissolving formulation, in which a marked increase in needle interspacing did not lead to an improvement in insertion depth. Nevertheless, it should be noticed that the aspect ratio of D6 MN is slightly smaller than that of the D1 ones, which may also have an influence in these results. Other authors have reported an influence of needle spacing in MN array insertion and skin permeability (43), however in our study this parameter was not as relevant, most likely due to the higher importance of other parameters previously discussed such as needle height, shape and sharpness.

One of the aims of this work was to design a variety of MN arrays that would allow us to achieve a balance between satisfactory mechanical and insertion properties, and optimal drug loading and deposition in skin. An example of how this balance is not always achieved can be observed in the results obtained with designs D3 and D5. Despite showing high drug loading and comparable insertion properties, the amount of CAB Na delivered using D3 was only approximately 67% of that achieved with D2, and an even lower number was obtained when comparing D5 and D1 (43%). This is likely related with the different shape of these needles, with D3 and D5 MN arrays likely being more strongly affected than D2 and D1 by the skin elastic behaviour, which promotes the expulsion of the needle tips and therefore a higher deposition of drug at the skin surface. It is likely that increasing the insertion force used with these designs would lead to higher amounts of drug being deposited in the skin.

In general, drug deposition results correlated well with insertion depth, as expected, with designs D1 and D2 showing the highest values in both studies. A deeper insertion of needle tips into the skin is more likely to promote the deposition of the drug in those regions of the skin and not on the skin surface where it would be wasted by cleaning. When considering the influence of needle interspacing in this process, we could observe that despite having a poorer drug loading result, design D4 showed a relatively good level of drug deposition. The total amount of drug delivered to the skin was lower than that of D3, due to the reduced total number of needles in D4, but the efficiency of this delivery was higher. Finally, in the case of D6, drug loading was lower possibly due to the increased needle interspacing, which reduced the total number of needles per MN array. Similarly, the increased needle height in this design may have limited the drug-loaded formulation from filling a larger proportion of the needle tip by pressure-driven casting. Nevertheless, the same design allowed for a relatively good level of drug deposition in skin since that additional needle height probably allows for a deeper deposition of higher amounts of the loaded drug.

When considering HFMN arrays, their potential to address the transdermal delivery of low potency drugs, when combined with lyophilised wafer-like drug reservoirs, should be highlighted (29). For this purpose, we selected IBU Na as a model compound for in vitro permeation studies with HFMN. This formulation showed fast and significant swelling properties, which are particularly important to allow a rapid dissolution of drug-containing reservoirs applied on top of the MN array, facilitating drug diffusion through the array and into the skin (29). Both types of HFMN designs allowed a controlled transdermal permeation of IBU Na throughout a period of 24 h. However, while the conical MN array (D6) allowed the permeation of approximately 32 ± 8% of the initial drug amount, which is in line with previously published data (29), the cross-shaped one (D7) only led to a delivery of 9 ± 4% of the initial IBU Na load. In this case, the characteristic plateau observed at the latest time points of most drug permeation studies was not achieved, possibly explaining the lower amount of drug delivered with this MN array. It is also interesting to note that in the case of this HFMN array design the well described “burst effect” present in most drug permeation studies seems to be somehow delayed, happening only approximately 1 h after the beginning of the study. The shape and volume of these needles may explain a slower absorption of liquid upon insertion. The individual volume (in dry state) of one cross-shaped needle is approximately 0.099 mm^3^, while that of a conical needle is approximately 0.085 mm^3^. Together with its complex shape, this feature may increase the time required to fully saturate the swollen array with the drug before it can be permeated to the acceptor compartment.

## Conclusions

We have described a comprehensive approach to fabricate high-quality MN array master templates using 2PP 3D printing. The synergistic combination of design and printing optimisation allowed us to develop a flexible fabrication method that produced high quality and mechanically strong master templates within a short period of time. Without requiring complex and customised processing steps, this technology offers a high level of flexibility, allowing direct and easy manufacturing of MN array structures even for those without microfabrication expertise. In terms of academic and industrial research, this could help reduce the time spent on prototyping and initial screening, as researchers would be able to develop and test innovative MN array designs in a short timeframe, quickly testing different alternatives. As shown by the results obtained in this study, master templates obtained by 2PP 3D printing allowed the manufacturing of multiple MN array moulds, which were subsequently used to prepare polymeric MN arrays with promising results in terms of insertion, drug loading and drug delivery to the skin. The flexibility and simplicity of this template fabrication method was key to facilitate the study of the impact of different MN array design parameters in the final performance of MN arrays as drug delivery systems.

## Electronic supplementary material

ESM 1 (PDF 176 kb)
