# Supplementary material for: Two-Photon Polymerisation 3D Printing of Microneedle Array Templates with Versatile Designs: Application in the Development of Polymeric Drug Delivery Systems
Source: Pharm Res. 2020 Aug 27;37(9):174. doi: 10.1007/s11095-020-02887-9 (PMC7452932; doi:10.1007/s11095-020-02887-9)
Supplement: Supplementary file 1 — (PDF 176 kb) [file 11095_2020_2887_MOESM1_ESM.pdf]

Supplementary Figures

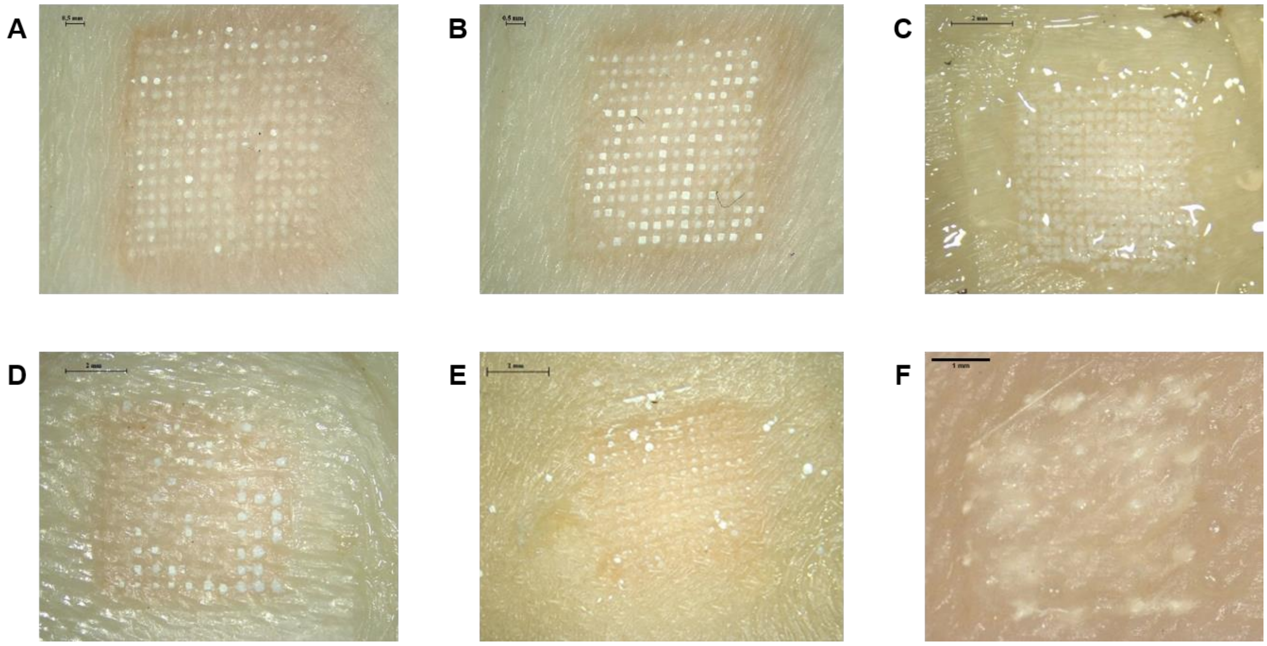

Figure S1. Light microscope images of neonatal porcine skin following insertion of CAB Na-loaded DMN arrays (**A**, D1 to **G**, D7).

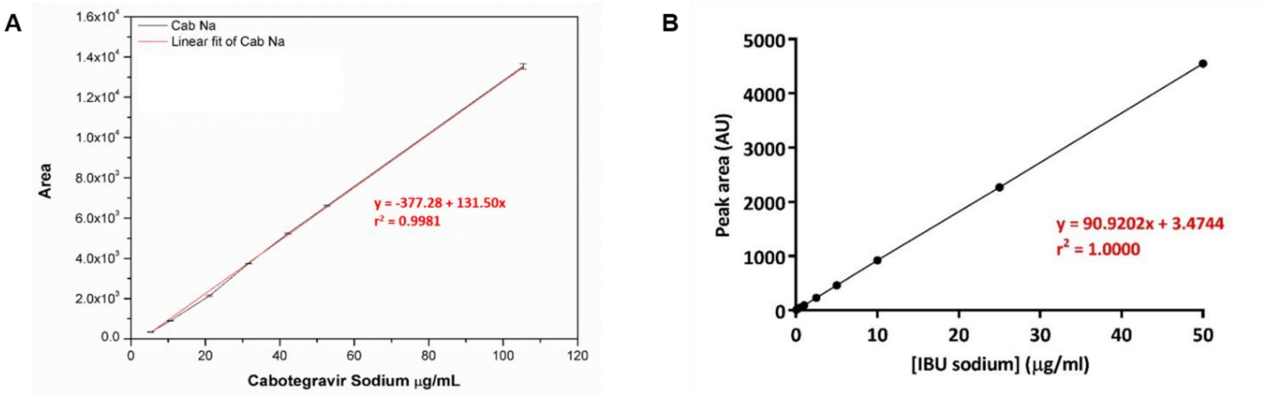

Figure S2. HPLC method validation: exemplar calibration curves obtained for **(A)** CAB Na and **(B)** IBU Na quantification.
